# Butterfly Glioma of the Corpus Callosum Mimicking Rapidly Progressive Dementia

**DOI:** 10.7759/cureus.111462

**Published:** 2026-06-25

**Authors:** Soumyadeep Maity, Girin Ray, Chandra Paul Gupta, Satyaki Basu, Kumar Kishlay

**Affiliations:** 1 General Medicine, KPC Medical College and Hospital, Kolkata, IND

**Keywords:** bpsd, butterfly glioma, corpus callosum tumour, dementia, glioma

## Abstract

A butterfly-pattern, high-grade glioma involving the corpus callosum is an uncommon but highly aggressive brain tumour that may present with neuropsychiatric manifestations and mimic rapidly progressive dementia, leading to diagnostic delay. Disruption of frontal-callosal connectivity may produce behavioural and cognitive abnormalities resembling behavioural and psychological symptoms of dementia (BPSD), resulting in initial misdiagnosis. A 65-year-old postmenopausal woman presented with a two-week history of vomiting, progressive disorientation, generalized weakness, irrelevant speech, cognitive decline, and bladder and bowel dysfunction. She had previously been treated for acute-onset dementia with BPSD using donepezil and memantine without prior neuroimaging. Neurological examination revealed asymmetric pyramidal signs, rigidity, and bilaterally constricted pupils. Arterial blood gas analysis showed respiratory alkalosis with hypocalcaemia, and urine culture isolated extended-spectrum beta-lactamase (ESBL)/AmpC-producing *Klebsiella aerogenes*. Contrast-enhanced MRI demonstrated a greater than 65 × 61 mm solid-cystic heterogeneously enhancing mass involving the genu of the corpus callosum with bifrontal extension and perilesional oedema, favouring a radiologically suspected high-grade glioma involving the corpus callosum. A stereotactic biopsy was planned for histopathological confirmation. The patient received corticosteroids, anti-epileptics, antibiotics, and supportive treatment, with subsequent clinical improvement. This case underscores the importance of early neuroimaging in atypical dementia presentations, especially when focal neurological deficits or signs of raised intracranial pressure are present. Histopathological confirmation remains essential for definitive diagnosis and treatment planning.

## Introduction

Glioblastoma multiforme (GBM) is the most common and most aggressive primary malignant brain tumour in adults and accounts for a substantial proportion of malignant primary central nervous system (CNS) neoplasms [1]. A particularly destructive pattern is the so-called butterfly variant, in which the lesion extends across the corpus callosum into both cerebral hemispheres and is associated with poor outcomes [2]. Involvement of the genu and anterior corpus callosum may disrupt frontal interhemispheric connectivity and produce prominent behavioural and cognitive symptoms, including apathy, disinhibition, executive dysfunction, irrelevant speech, and impaired self-care, thereby mimicking behavioural and psychological symptoms of dementia (BPSD) or other neurodegenerative syndromes [3,4]. This clinical overlap can result in initial psychiatric or dementia-focused evaluation, delayed neuroimaging, and inappropriate symptomatic treatment before a structural lesion is recognized. Recent case-based literature has emphasized that butterfly-pattern callosal lesions require careful diagnostic interpretation because imaging may strongly suggest high-grade glioma, but tissue diagnosis remains essential to distinguish GBM from other callosal neoplasms, including primary CNS lymphoma and related entities. For that reason, lesions of this type should be described as radiologically suspected high-grade glioma until stereotactic biopsy or histopathology confirms the diagnosis. We report the case of a 65-year-old woman who was initially managed as having acute-onset dementia with BPSD and treated with donepezil and memantine before contrast-enhanced MRI demonstrated a large bifrontal corpus callosum-crossing mass with radiological features suggestive of a high-grade glioma [5]. This case underscores the importance of early neuroimaging in atypical dementia presentations and highlights the need for multidisciplinary evaluation and diagnostic caution pending histopathological confirmation.

## Case presentation

A 65-year-old postmenopausal woman was brought by ambulance to the Emergency Department of a tertiary care medical college hospital with a two-week history of progressive drowsiness, disorientation, vomiting, irrelevant speech, generalized weakness, and loss of bladder and bowel control. She was unable to recognize familiar persons or places. Prior to admission, she had been evaluated by a psychiatrist and diagnosed with acute-onset dementia with BPSD and was started on donepezil and memantine without prior neuroimaging. As her symptoms continued to worsen, she was admitted for further evaluation. Her past medical history was significant for hypertension on treatment, with no history of malignancy, head trauma, seizures, or relevant family history. She was a non-smoker, did not consume alcohol, and was a housewife. On examination, she was drowsy but arousable, with a Glasgow Coma Scale (GCS) score of E4V4M5 (13/15). Vital signs were stable, with blood pressure of 100/60 mmHg, pulse rate of 97 beats per minute, temperature of 99.8°F, and oxygen saturation of 94% on room air. Random blood glucose was 156 mg/dL. Neurological examination demonstrated bilateral pupils that were constricted and sluggishly reactive to light. Tone was reduced in all four limbs; mild rigidity was also noted, likely reflecting the acute neurological state at presentation. The right plantar response was extensor, while the left plantar response was flexor, indicating asymmetric corticospinal tract involvement. These findings initially raised concern for a focal neurological lesion or cerebrovascular event, although precise hemispheric localization could not be reliably established clinically. Systemic examination was otherwise unremarkable. CBC and routine urine examination were normal. ABG demonstrated respiratory alkalosis with hypocalcaemia, the detailed values of which are presented (Table 1).

**Table 1:** Laboratory investigations at admission Initial laboratory parameters on admission showed mild anemia, respiratory alkalosis with hypocalcaemia, and a urine culture positive for *Klebsiella aerogenes *(extended-spectrum beta-lactamase (ESBL)/AmpC-producing organism). Other hematological, biochemical, and metabolic parameters were largely within normal limits. ALT: alanine transaminase; AST: aspartate aminotransferase

Parameter	Patient Value	Reference Range
Haemoglobin (g/dL)	11.9	12.0-16.0
Total leucocyte count (cells/µL)	9,600	4,000-11,000
Platelet count (cells/µL)	250,000	150,000–400,000
Urine culture	Klebsiella aerogenes (ESBL/AmpC)	No growth
Blood glucose - random (mg/dL)	156	70-140
Arterial pH	7.489	7.35-7.45
pCO₂ (mmHg)	31.7	35-45
HCO₃⁻ (mmol/L)	24.1	22-26
Serum Na⁺ (mmol/L)	141	136-145
Serum K⁺ (mmol/L)	3.5	3.5-5.0
Corrected Ca²⁺ (mmol/L)	1.1	1.15-1.35
Lactate (mmol/L)	0.9	0.5-2.2
Anion gap (mmol/L)	11	Aug-16
SpO₂ (%) on room air	94	95-100
AST (U/L)	16	0-31
ALT (U/L)	30	0-34

**Figure 1:**
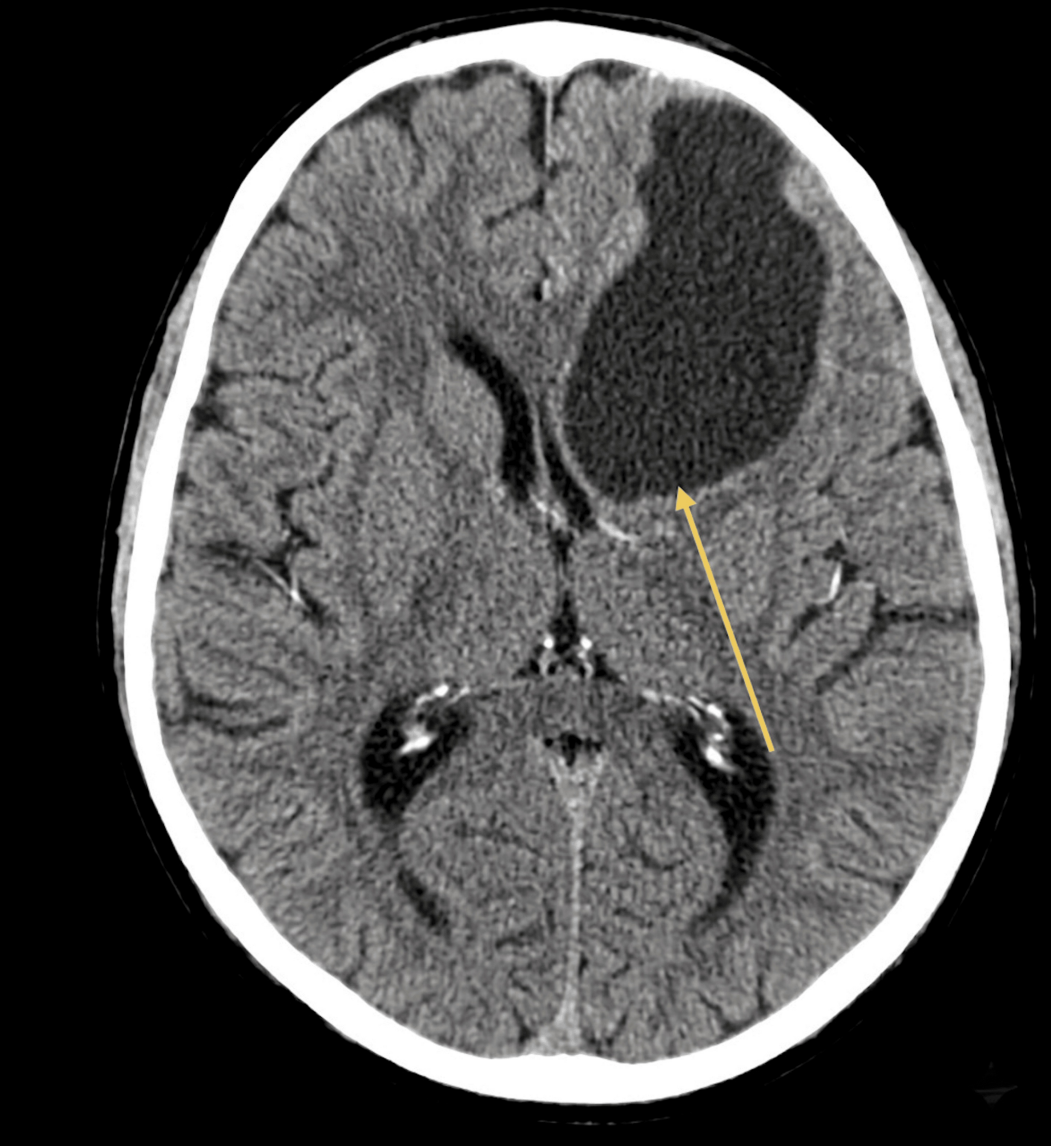
Non-contrast MRI of the brain showed a solid-cystic mass at the genu of the corpus callosum The yellow arrow points to the solid cystic mass lesion margin adjacent to the left lobe, compressing the nearby ventricle and midline shift.

Urine culture isolated *Klebsiella aerogenes *producing ESBL/AmpC enzymes, resistant to cephalosporins and fluoroquinolones but susceptible to carbapenems and cefoperazone-sulbactam, indicating a concurrent urinary tract infection that may have contributed to the acute deterioration. Non-contrast MRI of the brain demonstrated a moderately large solid-cystic lesion arising from the genu of the corpus callosum with bilateral frontal extension, surrounding perilesional oedema, and compression of the medial frontal structures (Figure 1). 

**Figure 2:**
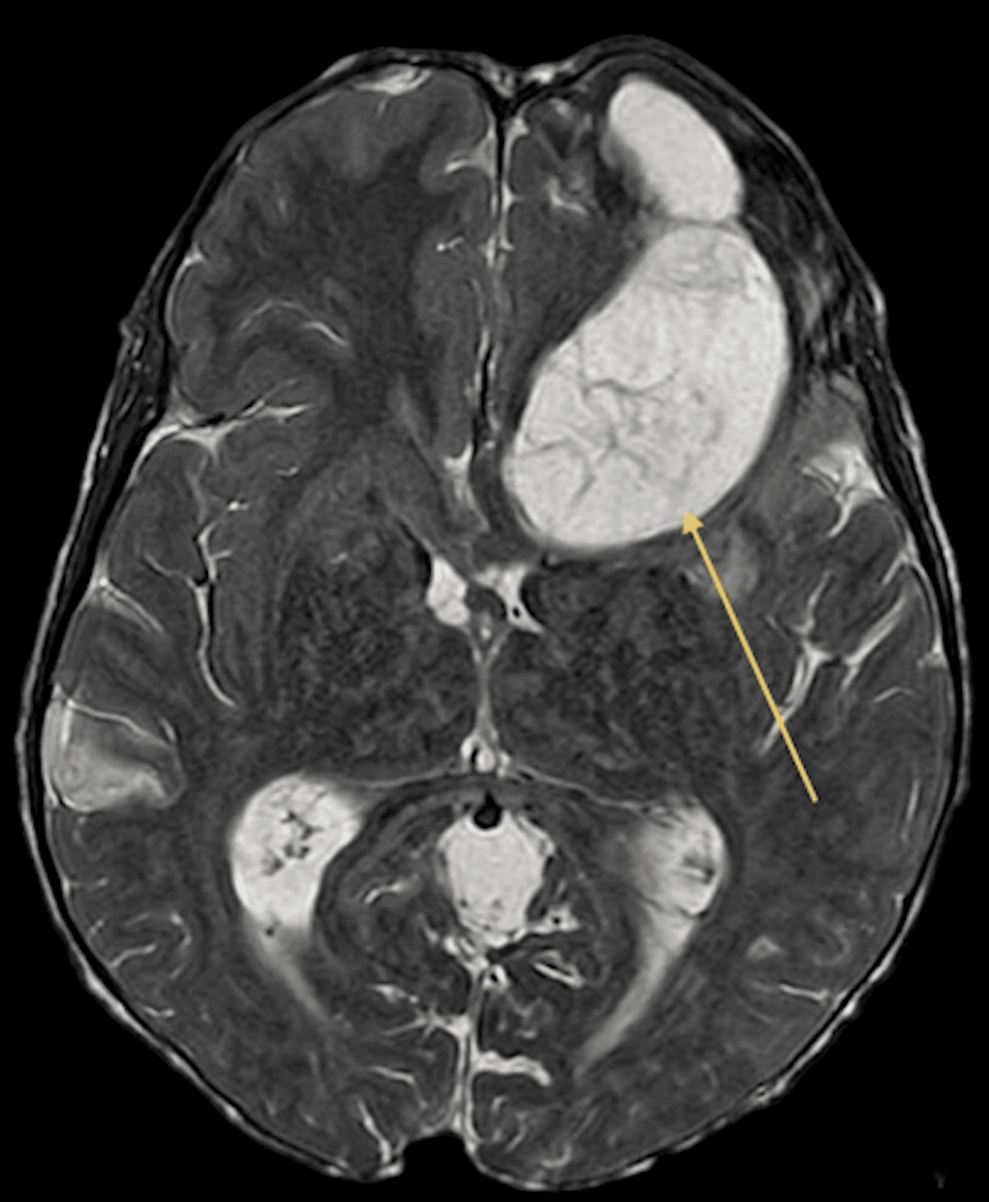
Contrast-enhanced MRI brain showing a hetero-intense bifrontal mass The yellow arrow points to the mass, projecting from the genu of the corpus callous with perilesional oedema and midline crossing.

The frontal horns of both lateral ventricles were effaced, and the cortical sulci were prominent. No posterior fossa, pituitary, or cranial nerve involvement was identified. Contrast-enhanced MRI of the brain, performed after intravenous gadolinium administration, confirmed a moderately large solid-cystic lesion projecting from the genu of the corpus callosum into the bifrontal region, measuring more than 65 × 61 mm on axial sections (Figure 2).

**Figure 3:**
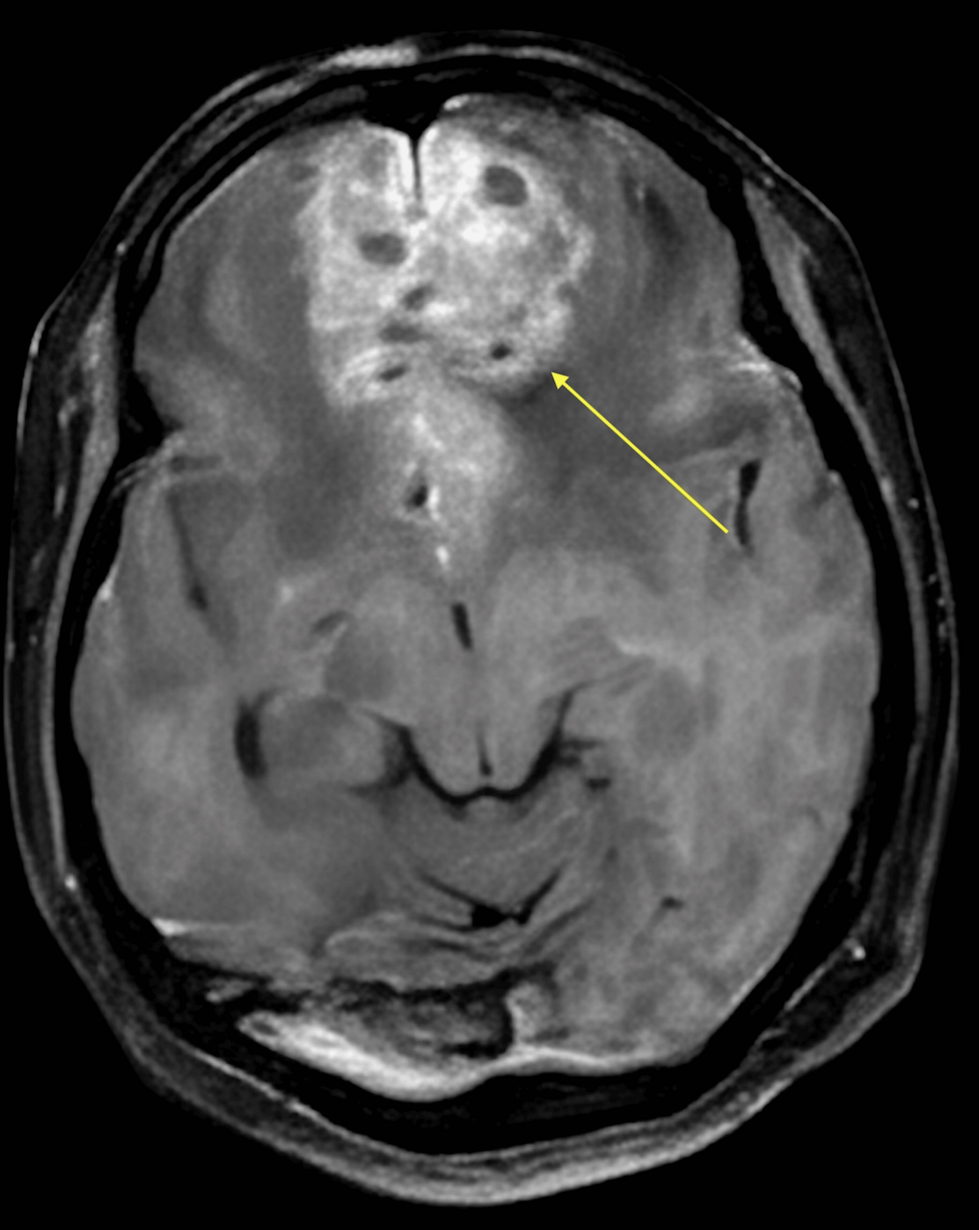
Contrast-enhanced axial MRI brain showing a heterogeneously enhancing solid-cystic mass crossing the genu of the corpus callosum with bifrontal extension and perilesional edema, suggestive of butterfly glioblastoma multiforme The yellow arrow indicates the enhancing lesion in the left frontal parasagittal region, with adjacent parenchymal compression and local mass effect.

The solid component demonstrated patchy heterogeneous enhancement. Perilesional oedema was seen in the bilateral periventricular white matter on T2/fluid-attenuated inversion recovery (FLAIR) sequences. The frontal horns of the lateral ventricles remained compressed, with the septum in the midline. The Sylvian fissures and extra-axial cerebrospinal fluid spaces were dilated. The radiological impression was of a moderately large solid-cystic lesion with surrounding oedema arising from the genu of the corpus callosum and involving both frontal lobes, favouring a high-grade neoplastic process, such as glioblastoma multiforme (Figure 3). 

The bifrontal midline-crossing mass with solid-cystic morphology and heterogeneous enhancement raised three principal differential diagnoses [6]. A butterfly-pattern high-grade glioma was considered the leading possibility, supported by the irregular heterogeneous enhancement, central cystic and necrotic components, perilesional oedema, and corpus callosum crossing, a pattern seen in aggressive neoplasms. Parasagittal or parafalcine meningioma was considered because of the bifrontal location and proximity to the midline; however, the absence of dural attachment and the presence of corpus callosum infiltration made this diagnosis less likely. Primary CNS lymphoma was also included in the differential diagnosis because it may present with rapidly progressive dementia and corpus callosum lesions, but it was considered less likely in view of the heterogeneous enhancement and internal cystic change, which are atypical imaging features for lymphoma. Following MRI, the patient was started on intravenous dexamethasone 8 mg twice daily to reduce cerebral oedema and relieve symptoms of raised intracranial pressure. Levetiracetam 500 mg twice daily was initiated for seizure prophylaxis. Carbapenem therapy was administered for the ESBL-producing *K. aerogenes *urinary tract infection in accordance with current guidance for resistant Gram-negative infections [7]. Calcium supplementation was provided for documented hypocalcaemia. The patient was referred to neurosurgical oncology for further evaluation. Stereotactic biopsy was planned for histopathological confirmation, including assessment of GFAP expression, IDH1/2 mutation status, O6-methylguanine-DNA methyltransferase (MGMT) promoter methylation, and Ki-67 proliferation index. A multidisciplinary discussion involving neurosurgery, neuro-oncology, and radiation oncology was recommended for treatment planning pending tissue diagnosis. During hospitalization, the patient showed gradual clinical improvement after corticosteroids, antiseizure therapy, treatment of the urinary tract infection, and correction of hypocalcaemia. At discharge, she was alert, conscious, and cooperative, with improved orientation and a marked reduction in irrelevant speech. She was counselled regarding the imaging findings, the need for stereotactic biopsy, and further oncological evaluation, and close follow-up with the neurosurgery and neuro-oncology teams was arranged.

## Discussion

This case exemplifies a diagnostically challenging presentation of a butterfly-pattern, high-grade glioma masquerading as BPSD-associated dementia. The patient's two-week history of progressive cognitive decline, irrelevant speech, disorientation, and sphincter disturbance prompted psychiatric evaluation and initiation of donepezil and memantine without neuroimaging, reflecting a well-recognized diagnostic pitfall in which corpus callosum neoplasms are misinterpreted as neurodegenerative dementia for weeks to months before the correct diagnosis is established [3-5]. Butterfly-pattern lesions involving the corpus callosum account for a small but clinically important subset of high-grade gliomas and are associated with prominent cognitive impairment at presentation. The bilateral frontal involvement through callosal genu infiltration explains the patient's behavioural and cognitive phenotype. The genu connects the prefrontal cortices bilaterally, and disruption of this network may impair executive function, emotional regulation, and higher cognitive processing, thereby producing symptoms that resemble BPSD [3]. The asymmetric plantar responses indicated corticospinal tract involvement, but precise hemispheric lateralization could not be reliably inferred clinically because the lesion was a midline-crossing bifrontal corpus callosum mass. Initial concern for a cerebrovascular event was therefore appropriately revised after MRI demonstrated a space-occupying lesion rather than an infarct pattern. The concurrent ESBL *K. aerogenes *urinary tract infection and hypocalcaemia (corrected Ca²⁺: 1.10 mmol/L) likely contributed to acute encephalopathic decompensation superimposed on the underlying intracranial pathology [8,9]. Contrast-enhanced MRI showing heterogeneous ring-like enhancement, central necrosis, solid-cystic morphology, and midline crossing through the corpus callosum is suggestive of a high-grade glioma [3,6]. Although primary CNS lymphoma was considered in the differential diagnosis, it was considered less likely because it typically shows homogeneous enhancement and less often demonstrates internal cystic change. Butterfly tumour series by Tunthanathip et al. has also shown that other histologies, including diffuse large B-cell lymphoma, may occur in this location, reinforcing the importance of tissue diagnosis in all callosal lesions [10]. Stereotactic biopsy with molecular characterization remains the gold standard for definitive diagnosis. Evaluation of MGMT promoter methylation and IDH1/2 status is essential for prognostication and classification under the 2021 WHO CNS tumour framework, in which IDH-mutated tumours are classified separately from glioblastoma [8,9]. Until histopathological confirmation is obtained, the lesion should be regarded as a radiologically suspected high-grade glioma rather than a confirmed glioblastoma. In suitable patients, the Stupp protocol with concurrent and adjuvant temozolomide and 60 Gy radiotherapy in 30 fractions remains the standard therapeutic approach for confirmed GBM, but final treatment planning should follow tissue diagnosis.

## Conclusions

A butterfly-pattern lesion involving the genu of the corpus callosum is a rare but important cause of rapidly progressive cognitive and behavioural decline and may closely mimic BPSD-associated dementia. This case highlights the risk of psychiatric misdiagnosis and delay in structural evaluation when neuroimaging is deferred in atypical dementia presentations. Elderly patients with subacute cognitive decline, behavioural disturbance, focal neurological signs, or symptoms of raised intracranial pressure, particularly vomiting and headache, should undergo urgent contrast-enhanced MRI. A bifrontal solid-cystic heterogeneously enhancing mass crossing the corpus callosum should be regarded as a radiologically suspected high-grade neoplasm until histopathological confirmation is obtained. Early multidisciplinary involvement of neurosurgery, neuro-oncology, radiation oncology, and infectious disease, together with correction of metabolic derangements and treatment of concurrent infections, is essential for optimal management.
